# The Effect of Admission During the Weekend On In-Hospital Outcomes for Patients With Peripartum Cardiomyopathy

**DOI:** 10.7759/cureus.31401

**Published:** 2022-11-12

**Authors:** Jose L Batista, Gustavo Duarte, Jose Mario Acosta Rullan, Nadia G Obaed, Daniel Karpel, Ambar Sekulits, Justin D Mark, Luis C Arcay, Rosario Colombo, Bryan Curry

**Affiliations:** 1 Internal Medicine, Hospital Corporation of America (HCA) Florida Aventura Hospital, Aventura, USA; 2 Internal Medicine, Albert Einstein College of Medicine, Jacobi Medical Center, New York, USA; 3 Medical School, Dr. Kiran C. Patel College of Allopathic Medicine, Nova Southeastern University, Fort Lauderdale, USA; 4 Cardiovascular Disease, Hospital Corporation of America (HCA) Florida Aventura Hospital, Aventura, USA; 5 Cardiovascular Division, University of Miami Miller School of Medicine/Jackson Memorial Hospital, Miami, USA

**Keywords:** cardiogenic shock, national inpatient sample, : peripartum cardiomyopathy, pregnancy, weekend effect

## Abstract

Background

Previous studies have shown that patients with heart failure (HF) and cardiogenic shock (CS) have worse outcomes when admitted over the weekend. Since peripartum cardiomyopathy (PPCM) is a cause of CS and persisting HF, it is reasonable to extrapolate that admission over the weekend would also have deleterious effects on PPCM outcomes. However, the impact of weekend admission has not been specifically evaluated in patients with PPCM.

Methods

We analyzed the National Inpatient Sample (NIS) from 2016 to 2019. The International Classification of Diseases, tenth revision (ICD-10) codes were used to identify all admissions with a primary diagnosis of PPCM. The sample was divided into weekday and weekend groups. We performed a multivariate regression analysis to estimate the effect of weekend admission on specified outcomes.

Results

A total of 6,120 admissions met the selection criteria, and 25.3% (n=1,550) were admitted over the weekend. The mean age was 31.3 ± 6.4 years. There were no significant differences in baseline characteristics between study groups. After multivariate analysis, weekend admission for PPCM was not associated with in-hospital mortality, ventricular arrhythmias, sudden cardiac arrest, thromboembolic events, cardiovascular implantable electronic device placement, and mechanical circulatory support insertion.

Conclusion

In conclusion, although HF and CS have been associated with worse outcomes when admitted over the weekend, we did not find weekend admission for PPCM to be independently associated with worse clinical outcomes after multivariate analysis. These findings could reflect improvement in the coordination of care over the weekend, improvement in physician handoff, and increased utilization of shock teams.

## Introduction

Peripartum cardiomyopathy (PPCM) is defined as an idiopathic cardiomyopathy occurring during the third trimester of pregnancy or within five months following delivery, abortion, or miscarriage, characterized by a reduced left ventricular ejection fraction <45%, and without other identifiable causes of heart failure (HF) [1-3]. Maternal death in PPCM is usually the result of ventricular arrhythmias, sudden cardiac arrest, thromboembolic events, and persistent or progressive HF resulting in cardiogenic shock [4]. Management is similar to standard HF therapy with particular considerations regarding medication side effects on the fetus, arrhythmia management, anticoagulation therapy, and mechanical circulatory support (MCS) [3]. Although partial or complete recovery of left ventricular function is common, PPCM has been shown to have increased morbidity and mortality in both the mother and the fetus [5-7].

Previous observational studies have shown that patients with HF and cardiogenic shock have worse outcomes when admitted over the weekend [8-11]. This is hypothesized to be related to limited staffing and resources over the weekend compared to the weekday. Moreover, significant delays in the care of patients with cardiogenic shock may spiral into rapid decompensation, organ failure, and death [12]. Since PPCM is a cause of cardiogenic shock and persistent HF, it is reasonable to extrapolate that admission over the weekend would also have deleterious effects on PPCM outcomes. However, the impact of weekend admission has not been specifically evaluated in patients with PPCM.

We sought to determine if admission during the weekend compared to admission during the weekday is associated with different clinical outcomes in adult females admitted for PPCM in the United States from 2016 to 2019.

## Materials and methods

The National Inpatient Sample (NIS) forms part of the Healthcare Cost and Utilization Project (HCUP) and is supported by the Agency for Healthcare Research and Quality (AHRQ). The NIS records all hospitalizations in 48 states plus the District of Columbia and thus represents 98% of the United States population. Weighted, the NIS estimates more than 35 million hospitalizations per year nationally [13]. The International Classification of Diseases, 10th edition (ICD-10) codes were used to identify the diagnosis and procedures performed [14]. The principal author acquired this data via the HCUP. Institutional Review Board approval was pursued but not required due to the publicly available nature of this de-identified limited database as compliant with the Health Insurance Portability and Accountability Act.

We analyzed the NIS from January 1, 2016, to December 31, 2019, to extract a cohort of all adult admissions (>18 years of age) with a primary diagnosis of PPCM using the ICD-10 code O90.3. We excluded those admissions who underwent percutaneous coronary intervention and coronary artery bypass graft during the index hospitalization to enhance the accuracy of the diagnosis. The NIS contains information on patients' demographics, socioeconomic status, hospital type, hospital size, length of stay at the hospital, and several other variables) [15]. The remainder of the clinical variables, including peripartum cardiomyopathy, transthoracic echocardiogram, percutaneous coronary intervention, coronary artery bypass graft, diabetes mellitus, etc, were identified using the ICD-10 codes (Table in Appendices) [14]. The Elixhauser comorbidity index (a well-validated score that uses 31 comorbid diagnoses to estimate intra-hospital mortality) was computed as a separate variable [16]. A list of the variables and their respective ICD-10 codes used in the study can be found in the Table in the Appendices section.

The primary endpoint was death due to any cause during the index admission and hospital length of stay. Secondary endpoints included ventricular arrhythmias, sudden cardiac arrest, thromboembolic events, placement of a cardiovascular implantable electronic device (CIED), and short-term MCS insertion during the index admission. Thromboembolic events were defined as a diagnosis of embolic stroke, intracardiac thrombus, or arterial embolism. CIED placement was defined as the placement of cardiac resynchronization therapy, implantable cardioverter defibrillator, or permanent pacemaker during the index admission. MCS was defined as the insertion of an intra-aortic balloon pump, Impella (Abiomed, Danvers, Massachusetts, United States), and extra-corporeal machine oxygenation (ECMO) during the index admission.

Weighted data were obtained by using the weight variable (DISCWT) that is provided by the HCUP. All statistical analyses were performed using weighted data. Values that were missing were excluded from the analysis. The Chi-square test was used to compare categorical variables, and these were described using frequency with percentages. The student's t-test was used for comparing continuous variables if normally distributed and reported as mean (± SD). The Mann-Whitney U test was used for comparing continuous variables with a skewed distribution, and they were reported as median (interquartile range [IQR]). Multivariable logistic and linear regression analysis was executed to estimate the relationship of weekend admission with all specified outcomes. The variables used for the construction of the regression model were either chosen from the database as existing variables or created with the use of ICD-10 codes or from the Elixhauser comorbidity index. The variables utilized for the regression model include the year of admission, sex, age, race/ethnicity, primary expected payer, household income, location of the hospital, teaching status of the hospital, pre-eclampsia, eclampsia, post-partum hemorrhage, uncomplicated diabetes, complicated diabetes, hepatic disease, coagulation disorders, fluid and electrolyte disturbances, pulmonary hypertension, chronic heart failure, chronic kidney disease, essential hypertension, complicated hypertension, peripheral vascular disease, hyperlipidemia, chronic obstructive pulmonary disease, body mass index ≥25 kg/m2, coronary artery disease, valvular pathology, hypothyroidism, hyperthyroidism, human immunodeficiency virus infection, underweight, malnutrition, obstructive sleep apnea, prior stroke, cancer in situ, metastatic cancer, psychotic disorders, tobacco usage, alcohol use, use of illicit drugs, and the Elixhauser comorbidity index. Variables were elected into the multivariate model if they reached the specified significance (p<0.20) in the univariate analysis. Besides, we forced variables known to be associated with the outcome. Logistic regression results are represented as adjusted odds ratios (ORs) and their respective 95% Confidence Intervals (CIs). Linear regression results are expressed as beta coefficients (Coef.) and their respective 95% CIs. Statistical analysis was performed using STATA/BE 17.0 (StataCorp, College Station, Texas, United States). P-values <0.05 were considered statistically significant. The checklist for working with the NIS was used to ensure the appropriateness of data analysis as recommended by AHRQ [17].

## Results

**Figure 1:**
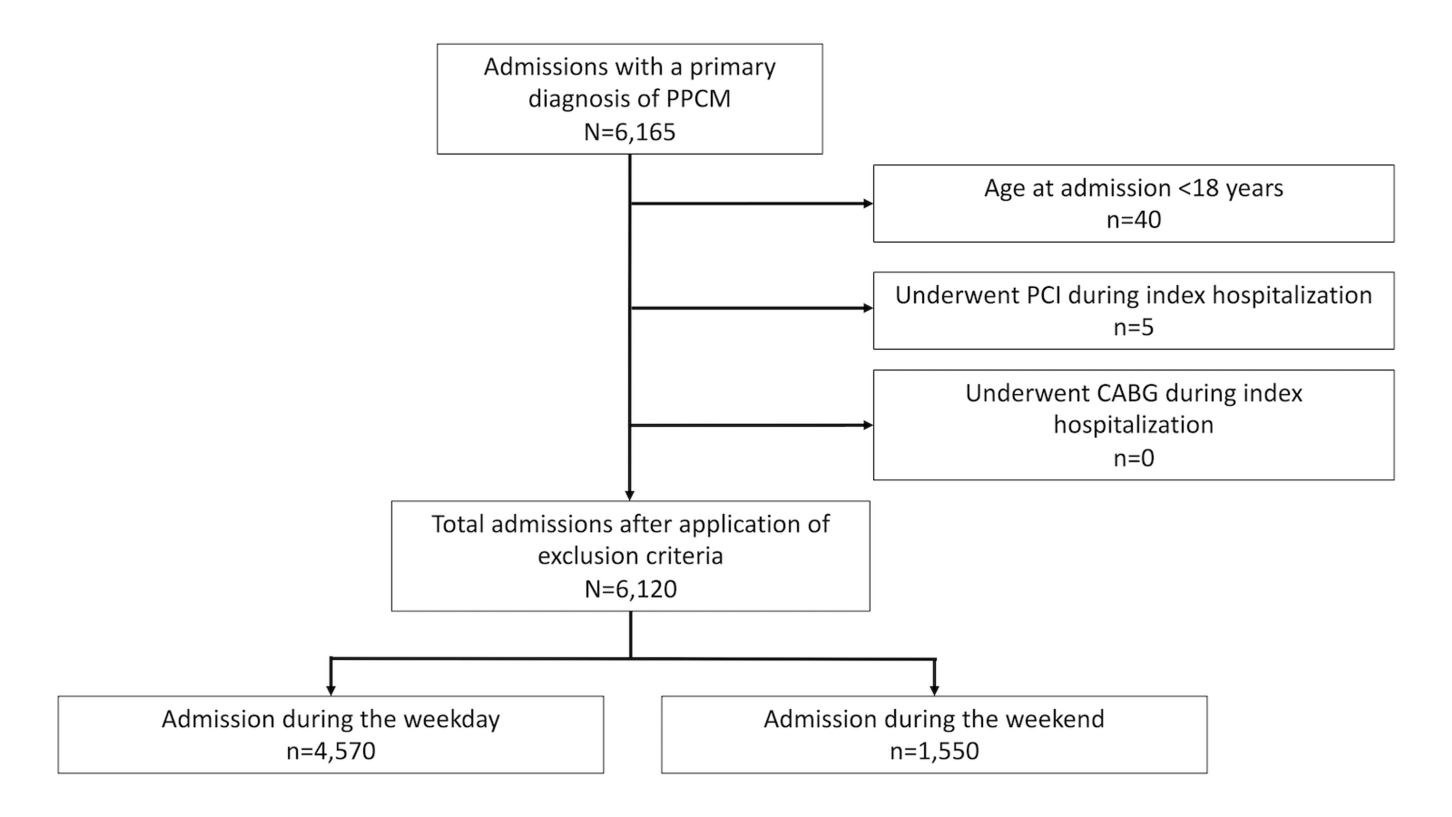
Flowchart showcasing participant selection criteria. PPCM, Peripartum Cardiomyopathy. PCI, Percutaneous Coronary Intervention. CABG, Coronary Artery Bypass Graft

We found a total of 6,165 hospitalizations with PPCM. Of these, only 6,120 admissions were included in the final analysis after exclusion criteria were applied (Figure 1).

**Figure 2:**
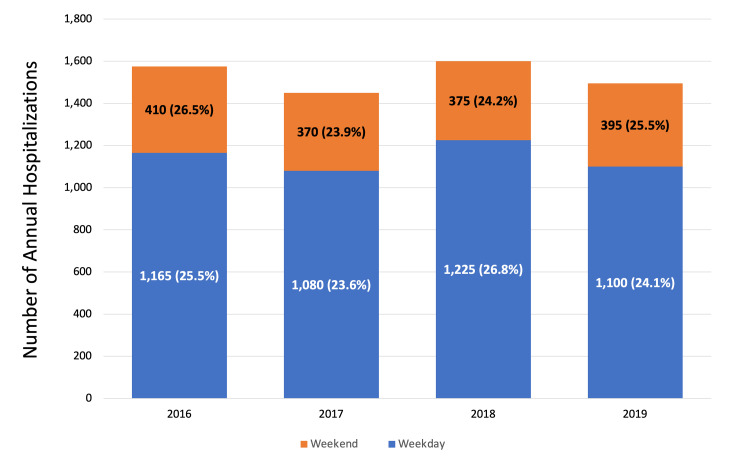
Trends in prevalence of weekday versus weekend admission of nationwide hospitalizations for peripartum cardiomyopathy.

The mean age of the sample was 31.3 ± 6.4 years. The number of PPCM hospitalizations remained the same during the study period, as seen in Figure 2.

As seen in Table 1, there were no significant differences in baseline characteristics among both study groups.

**Table 1:** Baseline characteristics of admissions for PPCM admitted during the weekday versus admitted over the weekend. For $n\leq 10$, the absolute numbers are not reported as per the Healthcare Cost and Utilization Project recommendations. ^a^There were 220 admissions missing race/ethnicity information; ^b^There were 195 admissions missing primary payer status information; ^c^Median household income national quartile for patient ZIP Code, there were 50 admissions missing income information; ^d^HELLP, Hemolysis, Elevated Liver Enzymes, and Low Platelets; ^e^BMI, Body Mass Index; PPCM, Peripartum Cardiomyopathy.

Baseline characteristics		Weekday (n= 4,570)	Weekend (n=1,550)	p-value
Age, years (Mean, SD)		31.2 (6.4)	31.5 (6.3)	<0.001
Race (n, %) ^a^				0.461
White		1,825 (41.1)	565 (38.6)	
Black		1,850 (41.7)	615 (42.0)	
Hispanic		400 (9.0)	190 (13.0)	
Asian or Pacific Islander		130 (2.9)	35 (2.4)	
Native American		60 (1.4)	15 (1.0)	
Other		170 (3.8)	45 (3.1)	
Calendar year (n, %)				0.834
2016		1,165 (25.5)	410 (26.5)	
2017		1,080 (23.6)	370 (23.9)	
2018		1,225 (26.8)	375 (24.2)	
2019		1,100 (24.1)	395 (25.5)	
Insurance type (n, %) ^b^				0.574
Medicare		180 (4.1)	65 (4.3)	
Medicaid		2,470 (55.9)	775 (51.5)	
Private insurance		1,655 (37.4)	615 (40.9)	
Self-Pay		115 (2.6)	50 (3.3)	
Teaching status (n, %)				0.726
Teaching		3,535 (77.4)	1,185 (76.5)	
Non-teaching		1,035 (22.6)	365 (23.5)	
Hospital location (n, %)				0.212
Rural		240 (5.3)	105 (6.8)	
Urban		4,330 (94.7)	1,445 (93.2)	
Median household income (n, %) ^c^				0.050
0-25th percentile		1,930 (42.6)	545 (35.4)	
26th-50th percentile		1,200 (26.5)	415 (26.9)	
51st-75th percentile		910 (20.1)	340 (22.1)	
76th-100th percentile		490 (10.8)	240 (15.6)	
Comorbidities (n, %)				
	Diabetes mellitus without complications	495 (10.8)	205 (13.2)	0.245
	Diabetes mellitus with complications	125 (2.7)	50 (3.2)	0.658
	Gestational diabetes	160 (3.5)	40 (2.6)	0.435
	Pre-eclampsia	595 (13.0)	210 (13.5)	0.813
	Eclampsia	55 (1.2)	£10 (£0.6)	0.405
	HELLP syndrome ^d^	£10 (£0.2)	£10 (£0.6)	0.422
	Hypertension, uncomplicated	305 (6.7)	60 (3.9)	0.073
	Hypertension, complicated	1,005 (22.0)	285 (18.4)	0.171
	Hyperlipidemia	£10 (£0.2)	15 (1.0)	0.075
	BMI ³25 Kg/m^2 e^	1,185 (25.9)	435 (28.1)	0.452
	Coronary artery disease	125 (2.7)	15 (1.0)	0.072
	Chronic heart failure	130 (2.8)	40 (2.6)	0.810
	Chronic kidney disease	125 (2.7)	55 (3.5)	0.458
	Valvular disease	915 (20.0)	315 (20.3)	0.907
	Hypothyroidism	250 (5.5)	60 (3.9)	0.257
	Liver disease	210 (4.6)	70 (4.5)	0.951
	Coagulopathy	140 (3.1)	80 (5.2)	0.076
	Underweight	20 (0.4)	£10 (£0.2)	0.243
	Malnutrition	55 (1.2)	£10 (£0.2)	0.277
	Ethanol	15 (0.3)	£10 (£0.2)	0.449
	Tobacco	610 (13.3)	185 (11.9)	0.525
	Illicit drugs	300 (6.6)	50 (3.2)	0.021

The all-cause in-hospital mortality rate for the study population was 0.8% (n=50), with no significant difference in all-cause in-hospital mortality among both study groups (p=0.693). The overall rate of ventricular arrhythmias was 5.6% (n=340), and sudden cardiac arrest occurred in 1.2% (n=75) admissions. Thromboembolic events occurred in 3.5% (n=205) of admissions. CIED placement occurred in 1.4% (n=85) of the study sample and MCS insertion in 2.9% (n=180). Nonetheless, there was no statistically significant difference in the rates of ventricular arrhythmias, sudden cardiac arrest, thromboembolic events, CIED placement, and MCS utilization among both study groups.

After multivariate adjustment, admission during the weekend was not independently associated with all-cause in-hospital mortality (OR=0.96, 95% CI 0.04-22.41, p=0.978), ventricular arrhythmias (OR=0.59, 95% CI 0.29-1.20, p=0.146), sudden cardiac arrest (OR=0.96, 95% CI 0.24-3.88, p=0.954), thromboembolic events (OR=0.86, 95% CI 0.36-2.02, p=0.724), CIED placement (OR=0.96, 95% CI 0.31-2.95, p=0.94), and insertion of a short-term MCS device (OR=1.14, 95% CI 0.41-3.28, p=0.809). The rates and the adjusted ORs for the aforesaid endpoints can be visualized in Table 2.

**Table 2:** Adjusted comparative outcomes among admissions for PPCM admitted over the weekday versus admitted over the weekend. For $n\leq 10$, the absolute numbers are not reported as per the Healthcare Cost and Utilization Project recommendations. Values are as n (%) unless otherwise indicated. PPCM, Peripartum Cardiomyopathy; SCA, Sudden Cardiac Arrest; CIED, Cardiovascular Implantable Electronic Device; MCS, Mechanical Circulatory Support; OR, Odds Ratio; CI, Confidence Interval

Outcomes	Weekday (n =4,570)	Weekend (n =1,550)	OR	95% CI	p value
All-cause mortality	40 (0.9)	<10 (<0.6)	0.96	0.04-22.41	0.978
Ventricular arrhythmias	280 (6.1)	60 (3.9)	0.59	0.29-1.20	0.146
SCA	60 (1.3)	15 (1.0)	0.96	0.24-3.88	0.954
Thromboembolic events	155 (3.4)	50 (3.2)	0.86	0.36-2.02	0.724
CIED placement	65 (1.4)	20 (1.3)	0.96	0.31-2.95	0.940
MCS	130 (2.8)	40 (2.6)	1.14	0.41-3.28	0.809

## Discussion

In this observational study with over 6000 admissions for PPCM, we evaluated the impact of admission during the weekend (“weekend effect”) on several in-hospital clinical outcomes. In contrast with prior registry data from 2004 to 2011, the annual rates of PPCM hospitalizations remained steady throughout the study period [18]. This finding is most likely explained by the decrease in birth rates in the United States since 2010 [19]. In addition, studies in patients with acute coronary syndromes and HF suggest that patients admitted over the weekend tend to be more ill and have more comorbidities [10,20]. In our study, all baseline characteristics were evenly distributed regardless of the day of admission. Therefore, any differences in hospital outcomes would likely result from differences in quality of care.

Previous observational studies have shown an association between weekend admissions and increased in-hospital mortality rate compared to weekday admissions [21-23]. This has been attributed to decreased nursing and ancillary service staffing, reduced physician-patient ratio, lack of the physician's familiarity with patients, and reduced level of healthcare staff experience on the weekends [21,24,25] In particular, discontinuity of care, defined as coverage of a patient by a physician from another team, has been associated with a higher incidence of preventable adverse events [25]. While the effect of weekend admissions has been shown to impact in-hospital outcomes in both admissions for HF and cardiogenic shock negatively, no study to date has studied the effect of weekend admissions on the PPCM subpopulation. We hypothesized that the combination of delay in care stemming from decreased staffing, a significant increase in the number of patients covered by the same physician, and the remarkably rapid deterioration seen in patients with PPCM would result in worse outcomes in PPCM admissions that occurred during the weekend. Interestingly, even though PPCM is a cause of HF and cardiogenic shock, there was no association between weekend admission and all-cause in-hospital mortality, ventricular arrhythmias, sudden cardiac arrest, thromboembolic events, CIED placement, and MCS insertion in PPCM hospitalizations.

Several factors could be responsible for these findings. Concerning the findings of all-cause in-hospital mortality, these are likely related to the study being underpowered since there were very few events of this outcome, and the confidence intervals were very wide. Other possibilities for the results of the other outcomes are increased recognition of the weekend effect and resultant improvement in the coordination and quality of care provided over the weekend. The weekend effect in cardiogenic shock and HF admissions was last studied in 2014 and 2017, respectively [8,11]. Therefore, it is possible that through the implementation of protocols for time-sensitive conditions, the gap in quality of care observed over the weekend has been either bridged or reduced. However, data regarding this particular question in the United States is lacking. Another possibility is that since PPCM is known to exhibit rapid deterioration, physicians caring for these patients over the weekend have a lower threshold for escalation of care and admission to levels of care with higher nurse-to-patient ratios. It may also be that efforts to improve the handoff of patients between physician shifts have led to a better quality of care by physicians taking care of patients over the weekend and, thus, fewer preventable adverse events. However, a recent study published in 2021 by Farid et al. [26] showed that physician handoff was only associated with slightly greater mortality among patients with high illness severity. Nonetheless, there is a paucity of contemporary data regarding physician handoff and preventable adverse events.

A final possibility is an increase in the implementation of “shock teams” throughout the United States. A shock team is a multidisciplinary team that facilitates early recognition and intervention of shock and expedites the release of resources [27]. Shock teams improve survival and increase utilization of invasive hemodynamic monitoring and advanced MCS devices while reducing overall MCS utilization [27,28]. Nonetheless, data regarding the adoption of shock team protocols in the United States is lacking.

This study must be interpreted in light of the limitations of administrative data. Information such as symptoms, laboratory results, vital signs, systolic function, medications, and functional capacity is unavailable. The accuracy of the diagnosis relies on the provider's coding, and some diagnoses may be miscoded or under-coded to a certain degree. In addition, the timing and sequence of secondary diagnoses cannot be assessed in relation to the hospitalization at hand. Procedures are reliably known to be performed during the index hospitalization. Furthermore, it is also crucial to remember that each observation in the NIS is not an individual patient but a hospitalization. This means that multiple observations could represent the same patient in the database, and patients cannot be tracked after discharge from the hospital.

## Conclusions

Although admissions for HF and cardiogenic shock have been associated with worse outcomes when admitted over the weekend, weekend admission was not independently associated with increased all-cause in-hospital mortality, hospital length of stay, ventricular arrhythmias, sudden cardiac arrest, thromboembolic events, CIED placement, and MCS insertion after multivariate analysis. These findings could be the reflection of several factors, such as improvement in coordination of care over the weekend, a lower threshold for escalation of care in this population, improvement in physician-to-physician transfer of care, and increased utilization of shock teams. However, data supporting these hypotheses are lacking. As such, the findings of this study suggest the need for re-evaluation of admission over the weekend in HF and cardiogenic shock patients to further evaluate if the same results can be reproduced or if this is only seen in the subpopulation of HF and cardiogenic shock patients with PPCM.

## Appendices

**Table 3:** ICD-10-CM/PCS codes used to identify diagnosis and procedures for clinical variables. ^a^ICD-10-CM/PCS, International Classification of Diseases, Tenth Revision, Clinical Modification and Procedure Classification System. ^b^CKD, Chronic Kidney Disease; ^c^PVD, Peripheral Vascular Disease; ^d^COPD, Chronic Obstructive Pulmonary Disease; ^e^HIV, Human Immunodeficiency Virus; ^f^CRTP, Cardiac Resynchronization Therapy with Pacemaker Function; ^g^CRTD, Cardiac Resynchronization Therapy with Defibrillator Function.

Variables	ICD-10-CM/PCS Codes ^a^
Peripartum Cardiomyopathy	O90.3
Transthoracic Echocardiogram	X2JAX47
Percutaneous Coronary Intervention (PCI)	02703, 02704, 02713, 02714, 02723, 02724, 02733, 02734, 02C03, 02C04, 02C13, 02C14, 02C23, 02C24, 02C33, 02C34
Diagnostic catheterization Without Intervention	B2000Z, B2001ZZ, B200YZZ, B2010ZZ, B2011ZZ, B201YZZ, B2020ZZ, B2021ZZ, B202YZZ, B2030ZZ, B2031ZZ, B203YZZ, B2040ZZ, B2041ZZ, B204YZZ, B2050ZZ, B2051ZZ, B2060ZZ, B2061ZZ, B206YZZ, B2070ZZ, B2071ZZ, B207YZZ, B2080ZZ, B2081ZZ, B208YZZ, B20F0ZZ, B20F1ZZ, B20FYZZ, B210010, B2100ZZ, B210110, B2101ZZ, B210Y10, B210YZZ, B211010, B2110ZZ, B211110, B2111ZZ, B211Y10, B211YZZ, B212010, B2120ZZ, B212110, B2121ZZ, B212Y10, B212YZZ, B213010, B2130ZZ, B213110, B2131ZZ, B213Y10, B213YZZ, B2140ZZ, B2141ZZ, B214YZZ, B2150ZZ, B2151ZZ, B215YZZ, B2160ZZ, B2161ZZ, B216YZZ, B2170ZZ, B2171ZZ, B217YZZ, B2180ZZ, B2181ZZ, B218YZZ, B21F0ZZ, B21F1ZZ, B21FYZZ
Coronary Artery Bypass Graft (CABG)	B202, B212, B203, B213, B223, B233, 02100Z3, 02110Z3, 02120Z3, 02100ZC, 02100ZF, 02110ZC, 02110ZF, 02120ZC, 02120ZF, 0210083, 0210093, 02100A3, 02100J3, 02100K3, 0210483, 0210493, 02104A3, 02104J3, 02104K3, 02104Z3, 0210344, 02103D4, 0210444, 02104D4, 0211083, 0211093, 02110A3, 02110J3, 02110K3, 0211483, 0211493, 02114A3, 02114J3, 02114K3, 02114Z3, 0212083, 0212093, 02120A3, 02120J3, 02120K3, 0212483, 0212493, 02124A3, 02124J3, 02124K3, 02124Z3, 0213083, 0213093, 02130A3, 02130J3, 02130K3, 02130Z3, 0213483, 0213493, 02134A3, 02134J3, 02134K3, 02134Z3
Diabetes Mellitus Without Chronic Complications	E08.00, E08.01, E08.10, E08.11, E08.9, E09.00, E09.01, E09.10, E09.11, E09.9, E10, E11, E13.00, O24.011, O24.012 O24.013, O24.019, O24.02, O24.03, O24.111, O24.112, O24.113, O24.119, O24.12, O24.13, O24.311, O24.312, O24.313, O24.319, O24.32, O24.33, O24.410, O24.414, O24.415, O24.419, O24.420, O24.424, O24.425, O24.429, O24.430, O24.434, O24.435, O24.439, O24.811, O24.812, O24.813, O24.819, O24.82, O24.83, O24.911, O24.912, O24.913, O24.919, O24.92, O24.93
Diabetes Mellitus with Chronic Complications	E08.21, E08.22, E08.29, E08.311, E08.319, E08.321, E08.3211, E08.3212, E08.3213, E08.3219, E08.329, E08.3291, E08.3292, E08.3293, E08.3299, E08.331, E08.3311, E08.3312, E08.3313, E08.3319, E08.339, E08.3391, E08.3392, E08.3393, E08.3399, E08.341, E08.3411, E08.3412, E08.3413, E08.3419, E08.349, E08.3491, E08.3492, E08.3493, E08.3499, E08.351, E08.3511, E08.3512, E08.3513, E08.3519, E08.3521, E08.3522, E08.3523, E08.3529, E08.3531, E08.3532, E08.3533, E08.3539, E08.3541, E08.3542, E08.3543, E08.3549, E08.3551, E08.3552, E08.3553, E08.3559, E08.359, E08.3591, E08.3592, E08.3593, E08.3599, E08.36, E08.37X1, E08.37X2, E08.37X3, E08.37X9, E08.39, E08.40, E08.41, E08.42, E08.43, E08.44, E08.49, E08.51, E08.52, E08.59, E08.610, E08.618, E08.620, E08.621, E08.622, E08.628, E08.630, E08.638, E08.641, E08.649, E08.65, E08.69, E08.8, E09.21, E09.22, E09.29, E09.311, E09.319, E09.321, E09.3211, E09.3212, E09.3213, E09.3219, E09.329, E09.3291, E09.3292, E09.3293, E09.3299, E09.331, E09.3311, E09.3312, E09.3313, E09.3319, E09.339, E09.3391, E09.3392, E09.3393, E09.3399, E09.341, E09.3411, E09.3412, E09.3413, E09.3419, E09.349, E09.3491, E09.3492, E09.3493, E09.3499, E09.351, E09.3511, E09.3512, E09.3513, E09.3519, E09.3521, E09.3522, E09.3523, E09.3529, E09.3531, E09.3532, E09.3533, E09.3539, E09.3541, E09.3542, E09.3543, E09.3549, E09.3551, E09.3552, E09.3553, E09.3559, E09.359, E09.3591, E09.3592, E09.3593, E09.3599, E09.36, E09.37X1, E09.37X2, E09.37X3, E09.37X9, E09.39, E09.40, E09.41, E09.42, E09.43, E09.44, E09.49, E09.51, E09.52, E09.59, E09.610, E09.618, E09.620, E09.621, E09.622, E09.628, E09.630, E09.638, E09.641, E09.649, E09.65, E09.69, E09.8, E10.21, E10.22, E10.29, E10.311, E10.319, E10.321, E10.3211, E10.3212, E10.3213, E10.3219, E10.329, E10.3291, E10.3292, E10.3293, E10.3299, E10.331, E10.3311, E10.3312, E10.3313, E10.3319, E10.339, E10.3391, E10.3392, E10.3393, E10.3399, E10.341, E10.3411, E10.3412, E10.3413, E10.3419, E10.349, E10.3491, E10.3492, E10.3493, E10.3499, E10.351, E10.3511, E10.3512, E10.3513, E10.3519, E10.3521, E10.3522, E10.3523, E10.3529, E10.3531, E10.3532, E10.3533, E10.3539, E10.3541, E10.3542, E10.3543, E10.3549, E10.3551, E103.552, E10.3553, E10.3559, E10.359, E10.3591, E10.3592, E10.3593, E10.3599, E10.36, E10.37X1, E10.37X2, E10.37X3, E10.37X9, E10.39, E10.40, E10.41, E10.42, E10.43, E10.44, E10.49, E10.51, E10.52, E10.59, E10.610, E10.618, E10.620, E10.621, E10.622, E10.628, E10.630, E10.638, E10.641, E10.649, E10.65, E10.69, E10.8, E11.21, E11.22, E11.29, E11.311, E11.319, E11.321, E11.3211, E11.3212, E11.3213, E11.3219, E11.329, E11.3291, E11.3292, E11.3293, E11.3299, E11.331, E11.3311, E11.3312, E11.3313, E11.3319, E11.339, E11.3391, E11.3392, E11.3393, E11.3399, E11.341, E11.3411, E11.3412, E11.3413, E11.3419, E11.349, E11.3491, E11.3492, E11.3493, E11.3499, E11.351, E11.3511, E11.3512, E11.3513, E11.3519, E11.3521, E11.3522, E11.3523, E11.3529, E11.3531, E11.3532, E11.3533, E11.3539, E11.3541, E11.3542, E11.3543, E11.3549, E11.3551, E11.3552, E11.3553, E11.3559, E11.359, E11.3591, E11.3592, E11.3593, E11.3599, E11.36, E11.37X1, E11.37X2, E11.37X3, E11.37X9, E11.39, E11.40, E11.41, E11.42, E11.43, E11.44, E11.49, E11.51, E11.52, E11.59, E11.610, E11.618, E11.620, E11.621, E11.622, E11.628, E11.630, E11.638, E11.641, E11.649, E11.65, E11.69, E11.8, E13.21, E13.22, E13.29, E13.311, E13.319, E13.321, E13.3211, E13.3212, E13.3213, E13.3219, E13.329, E13.3291, E13.3292, E13.3293, E13.3299, E13.331, E13.3311, E13.3312, E13.3313, E13.3319, E13.339, E13.3391, E13.3392, E13.3393, E13.3399, E13.341, E13.3411, E13.3412, E13.3413, E13.3419, E13.349, E13.3491, E13.3492, E13.3493, E13.3499, E13.351, E13.3511, E13.3512, E13.3513, E13.3519, E13.3521, E13.3522, E13.3523, E13.3529, E13.3531, E13.3532, E13.3533, E13.3539, E13.3541, E13.3542, E13.3543, E13.3549, E13.3551, E13.3552, E13.3553, E13.3559, E13.359, E13.3591, E13.3592, E13.3593, E13.3599, E13.36, E13.37X1, E13.37X2, E13.37X3, E13.37X9, E13.39, E13.40, E13.41, E13.42, E13.43, E13.44, E13.49, E13.51, E13.52, E13.59, E13.610, E13.618, E13.620, E13.621, E13.622, E13.628, E13.630, E13.638, E13.641, E13.649, E13.65, E13.69, E13.8
Liver Disease	A51.45, A52.74, B18.0, B18.1, B18.2, B18.8, B18.9, B19.0, B19.10, B19.11, B19.20, B19.21, B19.9, B25.1, B58.1, I85.00, I85.01, I85.10, I85.11, I86.4, K70.0, K70.10, K70.11, K70.2, K70.30, K70.31, K70.40, K70.41, K70.9, K71.3, K71.4, K71.50, K71.51, K71.6, K71.7, K71.8, K72.10, K72.11, K73.0, K73.1, K73.2, K73.8, K73.9, K74.0, K74.00, K74.01, K74.02, K74.1, K74.2, K74.3, K74.4, K74.5, K74.60, K74.69, K75.1, K75.2, K75.3, K75.4, K75.81, K75.89, K75.9, K76.0, K76.1, K76.2, K76.3, K76.4, K76.5, K76.6, K76.7 K76.81, K76.89, K76.9, K77, K91.82, Z94.4
Coagulopathy	D61.09, D61.1, D61.2, D61.3, D61.810, D61.811, D61.818, D61.82, D61.89, D61.9, D65, D66, D67, D68.0, D68.1, D68.2, D68.311, D68.312, D68.318, D68.32, D68.4, D68.8, D68.9, D69.1, D69.3, D69.41, D69.42, D69.49, D69.51, D69.59, D69.6, D69.8, D69.9, D75.82
Solid Tumor Without Metastasis	D00.00, D00.01, D00.02, D00.03, D00.04, D00.05, D00.06, D00.07, D00.08, D00.1, D00.2, D01.0, D01.1, D01.2, D01.3, D01.40, D01.49, D01.5, D01.7, D01.9, D02.0, D02.1, D02.20, D02.21, D02.22, D02.3, D02.4, D03.0, D03.10, D03.11, D03.111, D03.112, D03.12, D03.121, D03.122, D03.20, D03.21, D03.22, D03.30, D03.39, D03.4, D03.51, D03.52, D03.59, D03.60, D03.61, D03.62, D03.70, D03.71, D03.72, D03.8, D03.9, D04.0, D04.10, D04.11, D04.111, D04.112, D04.12, D04.121, D04.122, D04.20, D04.21, D04.22, D04.30, D04.39, D04.4, D04.5, D04.60, D04.61, D04.62, D04.70, D04.71, D04.72, D04.8, D04.9, D05.00, D05.01, D05.02, D05.10, D05.11, D05.12, D05.80, D05.81, D05.82, D05.90, D05.91, D05.92, D06.0, D06.1, D06.7, D06.9, D07.0, D07.1, D07.2, D07.30, D07.39, D07.4, D07.5, D07.60, D07.61, D07.69, D09.0, D09.10, D09.19, D09.20, D09.21, D09.22, D09.3, D09.8, D09.9 C00.0, C00.1, C00.2, C00.3, C00.4, C00.5, C00.6, C00.8, C00.9, C01, C02.0, C02.1, C02.2, C02.3, C02.4, C02.8, C02.9, C03.0, C03.1, C03.9, C04.0, C04.1, C04.8, C04.9, C05.0, C05.1, C05.2, C05.8, C05.9, C06.0, C06.1, C06.2, C06.80, C06.89, C06.9, C07, C08.0, C08.1, C08.9, C09.0, C09.1, C09.8, C09.9, C10.0, C10.1, C10.2, C10.3, C10.4, C10.8, C10.9, C11.0, C11.1, C11.2, C11.3, C11.8, C11.9, C12, C13.0, C13.1, C13.2, C13.8, C13.9, C14.0, C14.2, C14.8, C15.3, C15.4, C15.5, C15.8, C15.9, C16.0, C16.1, C16.2, C16.3, C16.4, C16.5, C16.6, C16.8, C16.9, C17.0, C17.1, C17.2, C17.3, C17.8, C17.9, C18.0, C18.1, C18.2, C18.3, C18.4, C18.5, C18.6, C18.7, C18.8, C18.9, C19, C20, C21.0, C21.1, C21.2, C21.8, C22.0, C22.1, C22.2, C22.3, C22.4, C22.7, C22.8, C22.9, C23, C24.0, C24.1, C24.8, C24.9, C25.0, C25.1, C25.2, C25.3, C25.4, C25.7, C25.8, C25.9, C26.0, C26.1, C26.9, C30.0, C30.1, C31.0, C31.1, C31.2, C31.3, C31.8, C31.9, C32.0, C32.1, C32.2, C32.3, C32.8, C32.9, C33, C34.00, C34.01, C34.02, C34.10, C34.11, C34.12, C34.2, C34.30, C34.31, C34.32, C34.80, C34.81, C34.82, C34.90, C34.91, C34.92, C37, C38.0, C38.1, C38.2, C38.3, C38.4, C38.8, C39.0, C39.9, C40.00, C40.01, C40.02, C40.10, C40.11, C40.12, C40.20, C40.21, C40.22, C40.30, C40.31, C40.32, C40.80, C40.81, C40.82, C40.90, C40.91, C40.92, C41.0, C41.1, C41.2, C41.3, C41.4, C41.9, C43.0, C43.10, C43.11, C43.111, C43.112, C43.12, C43.121, C431.22, C43.20, C43.21, C43.22, C43.30, C43.31, C43.39, C43.4, C43.51, C43.52, C43.59, C43.60, C43.61, C43.62, C43.70, C43.71, C43.72, C43.8, C43.9, C44.00, C44.09, C44.101, C44.102, C44.1021, C44.1022, C44.109, C44.1091, C44.1092, C44.131, C44.1321, C44.1322, C44.1391, C44.1392, C44.191, C44.192, C44.1921, C44.1922, C44.199, C44.1991, C44.1992, C44.201, C44.202, C44.209, C44.291, C44.292, C44.299, C44.300, C44.301, C44.309, C44.390, C44.391, C44.399, C44.40, C44.49, C44.500, C44.501, C44.509, C44.590, C44.591, C44.599, C44.601, C44.602, C44.609, C44.691, C44.692, C44.699, C44.701, C44.702, C44.709, C44.791, C44.792, C44.799, C44.80, C44.89, C44.90, C44.99, C45.0, C45.1, C45.2, C45.7, C45.9, C46.0, C46.1, C46.2, C46.3, C46.4, C46.50, C46.51, C46.52, C46.7, C46.9, C47.0, C47.10, C47.11, C47.12, C47.20, C47.21, C47.22, C47.3, C47.4, C47.5, C47.6, C47.8, C47.9, C48.0, C48.1, C48.2, C48.8, C49.0, C49.10, C49.11, C49.12, C49.20, C49.21, C49.22, C49.3, C49.4, C49.5, C49.6, C49.8, C49.9, C49.A0, C49.A1, C49.A2, C49.A3, C49.A4, C49.A5, C49.A9, C4A.0, C4A.10, C4A.11, C4A.111, C4A.112, C4A.12, C4A.121, C4A.122, C4A.20, C4A.21, C4A.22, C4A.30, C4A.31, C4A.39, C4A.4, C4A.51, C4A.52, C4A.59, C4A.60, C4A.61, C4A.62, C4A.70, C4A.71, C4A.72, C4A.8, C4A.9, C50.011, C50.012, C50.019, C50.021, C50.022, C50.029, C50.111, C50.112, C50.119, C50.121, C50.122, C50.129, C50.211, C50.212, C50.219, C50.221, C50.222, C50.229, C50.311, C50.312, C50.319, C50.321, C50.322, C50.329, C50.411, C50.412, C50.419, C50.421, C50.422, C50.429, C50.511, C50.512, C50.519, C50.521, C50.522, C50.529, C50.611, C50.612, C50.619, C50.621, C50.622, C50.629, C50.811, C50.812, C50.819, C50.821, C50.822, C50.829, C50.911, C50.912, C50.919, C50.921, C50.922, C50.929, C51.0, C51.1, C51.2, C51.8, C51.9, C52, C53.0, C53.1, C53.8, C53.9, C54.0, C54.1, C54.2, C54.3, C54.8, C54.9, C55, C56.1, C56.2, C56.3, C56.9, C57.00, C57.01, C57.02, C57.10, C57.11, C57.12, C57.20, C57.21, C57.22, C57.3, C57.4, C57.7, C57.8, C57.9, C58, C60.0, C60.1, C60.2, C60.8, C60.9, C61, C62.00, C62.01, C62.02, C62.10, C62.11, C62.12, C62.90, C62.91, C62.92, C63.00, C63.01, C63.02, C63.10, C63.11, C63.12, C63.2, C63.7, C63.8, C63.9, C64.1, C64.2, C64.9, C65.1, C65.2, C65.9, C66.1, C66.2, C66.9, C67.0, C67.1, C67.2, C67.3, C67.4, C67.5, C67.6, C67.7, C67.8, C67.9, C68.0, C68.1, C68.8, C68.9, C69.00, C69.01, C69.02, C69.10, C69.11, C69.12, C69.20, C69.21, C69.22, C69.30, C69.31, C69.32, C69.40, C69.41, C69.42, C69.50, C69.51, C69.52, C69.60, C69.61, C69.62, C69.80, C69.81, C69.82, C69.90, C69.91, C69.92, C70.0, C70.1, C70.9, C71.0, C71.1, C71.2, C71.3, C71.4, C71.5, C71.6, C71.7, C71.8, C71.9, C72.0, C72.1, C72.20, C72.21, C72.22, C72.30, C72.31, C72.32, C72.40, C72.41, C72.42, C72.50, C72.59, C72.9, C73, C74.00, C74.01, C74.02, C74.10, C74.11, C74.12, C74.90, C74.91, C74.92, C75.0, C75.1, C75.2, C75.3, C75.4, C75.5, C75.8, C75.9, C76.0, C76.1, C76.2, C76.3, C76.40, C76.41, C76.42, C76.50, C76.51, C76.52, C76.8, C7A.00, C7A.010, C7A.011, C7A.012, C7A.019, C7A.020, C7A.021, C7A.022, C7A.023, C7A.024, C7A.025, C7A.026, C7A.029, C7A.090, C7A.091, C7A.092, C7A.093, C7A.094, C7A.095, C7A.096, C7A.098, C7A.1, C7A.8, D46.9, E31.21, E31.22, E31.23
Metastatic Cancer	C77.0, C77.1, C77.2, C77.3, C77.4, C77.5, C77.8, C77.9, C78.00, C78.01, C78.02, C78.1, C78.2, C78.30, C78.39, C78.4, C78.5, C78.6, C78.7, C78.80, C78.89, C79.00, C79.01, C79.02, C79.10, C79.11, C79.19, C79.2, C79.31, C79.32, C79.40, C79.49, C79.51, C79.52, C79.60, C79.61, C79.62, C79.63, C79.70, C79.71, C79.72, C79.81, C79.82, C79.89, C79.9, C7B.00, C7B.01, C7B.02, C7B.03, C7B.04, C7B.09, C7B.1, C7B.8, C80.0
Pulmonary hypertension	I27.0, I27.2, I27.20, I27.21, I27.22, I27.23, I27.24, I27.29
CKD ^b^	N18.1, N18.2, N18.3, N18.4, N18.5, N18.6, N18.9, I12.0, I13.11, I13.2, Z49, Z94.0, Z91.15, Z99.2
PVD ^c^	I70.0, I70.1, I70.201, I70.202, I70.203, I70.208, I70.209, I70.211, I70.212, I70.213, I70.218, I70.219, I70.221, I70.222, I70.223, I70.228, I70.229, I70.231, I70.232, I70.233, I70.234, I70.235, I70.238, I70.239, I70.241, I70.242, I70.243, I70.244, I70.245, I70.248, I70.249, I70.25, I70.261, I70.262, I70.263, I70.268, I70.269, I70.291, I70.292, I70.293, I70.298, I70.299, I70.301, I70.302, I70.303, I70.308, I70.309, I70.311, I70.312, I70.313, I70.318, I70.319, I70.321, I70.322, I70.323, I70.328, I70.329, I70.331, I70.332, I70.333, I70.334, I70.335, I70.338, I70.339, I70.341, I70.342, I70.343, I70.344, I70.345, I70.348, I70.349, I70.35, I70.361, I70.362, I70.363, I70.368, I70.369, I70.391, I70.392, I70.393, I70.398, I70.399, I70.401, I70.402, I70.403, I70.408, I70.409, I70.411, I70.412, I70.413, I70.418, I70.419, I70.421, I70.422, I70.423, I70.428, I70.429, I70.431, I70.432, I70.433, I70.434, I70.435, I70.438, I70.439, I70.441, I70.442, I70.443, I70.444, I70.445, I70.448, I70.449, I70.45, I70.461, I70.462, I70.463, I70.468, I70.469, I70.491, I70.492, I70.493, I70.498, I70.499, I70.501, I70.502, I70.503, I70.508, I70.509, I70.511, I70.512, I70.513, I70.518, I70.519, I70.521, I70.522, I70.523, I70.528, I70.529, I70.531, I70.532, I70.533, I70.534, I70.535, I70.538, I70.539, I70.541, I70.542, I70.543, I70.544, I70.545, I70.548, I70.549, I70.55, I70.561, I70.562, I70.563, I70.568, I70.569, I70.591, I70.592, I70.593, I70.598, I70.599, I70.601, I70.602, I70.603, I70.608, I70.609, I70.611, I70.612, I70.613, I70.618, I70.619, I70.621, I70.622, I70.623, I70.628, I70.629, I70.631, I70.632, I70.633, I70.634, I70.635, I70.638, I70.639, I70.641, I70.642, I70.643, I70.644, I70.645, I70.648, I70.649, I70.65, I70.661, I70.662, I70.663, I70.668, I70.669, I70.691, I70.692, I70.693, I70.698, I70.699, I70.701, I70.702, I70.703, I70.708, I70.709, I70.711, I70.712, I70.713, I70.718, I70.719, I70.721, I70.722, I70.723, I70.728, I70.729, I70.731, I70.732, I70.733, I70.734, I70.735, I70.738, I70.739, I70.741, I70.742, I70.743, I70.744, I70.745, I70.748, I70.749, I70.75, I70.761, I70.762, I70.763, I70.768, I70.769, I70.791, I70.792, I70.793, I70.798, I70.799, I70.8, I70.90, I70.91, I70.92, I71.00, I71.01, I71.02, I71.03, I71.1, I71.2, I71.3, I71.4, I71.5, I71.6, I71.8, I71.9, I72.0, I72.1, I72.2, I72.3, I72.4, I72.5, I72.6, I72.8, I72.9, I73.89, I73.9, I74.01, I74.09, I74.10, I74.11, I74.19, I74.2, I74.3, I74.4, I74.5, I74.8, I74.9, I75.011, I75.012, I75.013, I75.019, I75.021, I75.022, I75.023, I75.029, I75.81, I75.89, I77.0, I77.1, I77.2, I77.3, I77.4, I77.5, I77.6, I77.70, I77.71, I77.72, I77.73, I77.74, I77.75, I77.76, I77.77, I77.79, I77.810, I77.811, I77.812, I77.819, I77.89, I77.9, I79.0, I79.1, I79.8, K55.1, Z95.820, Z95.828
COPD^ d^	J43, J44, J41.0, J41.1, J41.8, J42
Chronic Heart Failure	I50.22, I50.32, I50.42
Hypertension, Complicated	I11.0, I13.0, I13.2
Primary Hypertension Uncomplicated	I10
Hyperlipidemia	E78.2, E78.4
Body mass index ³25 kg/m^2^	E66.01, E66.09, E66.2, E66.3, E66.8, E66.9, Z68.25, Z68.26, Z68.27, Z68.28, Z68.29, Z68.30, Z68.31, Z68.32, Z68.33, Z68.34, Z68.35, Z68.36, Z68.37, Z68.38, Z68.39, Z68.41, Z68.42, Z68.43, Z68.44, Z68.45
Carotid Artery Stenosis	I252, I249, I2510, I25110, I25111, I25118, I25119, I259, I2583
Coronary Artery Disease	I25.2, I24.9, I25.10, I25.111 I25.118, I25.119, I25.9, I25.83
Prior Stroke	I69.3, Z86.73
Hypothyroidism	E03.9, E89.0, E03.8, E03.0, E03.1, E03.2, E03.4, E03.5, E01.0, E01.1, E01.2, E01.8, E02
Hyperthyroidism	E0590
HIV ^e^	B20
Underweight	Z68.1, R63.6
Malnutrition	E43, E44.0, E44.1, E46
Tobacco Use	F17.203, F17.208, F17.209, F17.210, F17.211, F17.213, F17.218, F17.219, F17.220, F17.22, F17.223, F17.228, F17.290, F17.293, F19.298, F17.299
Ethanol	F10.10, F10.11, F10.120, F10.121, F10.129, F10.150, F10.151, F10.159, F10.180, F10.181, F10.182, F10.188, F10.19, F10.20, F10.21, F10.220, F10.221, F10.229, F10.230, F10.231, F10.232, F10.239, F10.24
Illicit Drugs	F11.10, F11.11, F11.120, F11.121, F11.122, F11.129, F11.13, F11.14, F11.150, F11.151, F11.159, F11.181, F11.182, F11.188, F11.19, F11.20, F11.21, F11.220, F11.221, F11.222, F11.229, F11.23, F11.24, F11.250, F11.251, F11.259, F11.281, F11.282, F11.288, F11.29, F12.10, F12.11, F12.120, F12.121, F12.122, F12.129, F12.13, F12.150, F12.151, F12.159, F12.180, F12.188, F12.19, F12.20, F12.21, F12.220, F12.221, F12.222, F12.229, F12.23, F12.250, F12.251, F12.259, F12.280, F12.288, F12.29, F13.10, F13.11, F13.120, F13.121, F13.129, F13.130, F13.131, F13.132, F13.139, F13.14, F13.150, F13.151, F13.159, F13.180, F13.181, F13.182, F13.188, F13.19, F13.20, F13.21, F13.220, F13.221, F13.229, F13.230, F13.231, F13.232, F13.239, F13.24, F13.250, F13.251, F13.259, F13.26, F13.27, F13.280, F13.281, F13.282, F13.288, F13.29, F14.10, F14.11, F14.120, F14.121, F14.122, F14.129, F14.13, F14.14, F14.150, F14.151, F14.159, F14.180, F14.181, F14.182, F14.188, F14.19, F14.20, F14.21, F14.220, F14.221, F14.222, F14.229, F14.23, F14.24, F14.250, F14.251, F14.259, F14.280, F14.281, F14.282, F14.288, F14.29, F15.10, F15.11, F15.120, F15.121, F15.122, F15.129, F15.13, F15.14, F15.150, F15.151, F15.159, F15.180, F15.181, F15.182, F15.188, F15.19, F15.20, F15.21, F15.220, F15.221, F15.222, F15.229, F15.23, F15.24, F15.250, F15.251, F15.259, F15.280, F15.281, F15.282, F15.288, F15.29, F16.10, F16.11, F16.120, F16.121, F16.122, F16.129, F16.14, F16.150, F16.151, F16.159, F16.180, F16.183, F16.188, F16.19, F16.20, F16.21, F16.220, F16.221, F16.229, F16.24, F16.250, F16.251, F16.259, F16.280, F16.283, F16.288, F16.29, F18.10, F18.11, F18.120, F18.121, F18.129, F18.14, F18.150, F18.151, F18.159, F18.17, F18.180, F18.188, F18.19, F18.20, F18.21, F18.220, F18.221, F18.229, F18.24, F18.250, F18.251, F18.259, F18.27, F18.280, F18.288, F18.29, F19.10, F19.11, F19.120, F19.121, F19.122, F19.129, F19.130, F19.131, F19.132, F19.139, F19.14, F19.150, F19.151, F19.159, F19.16, F19.17, F19.180, F19.181, F19.182, F19.188, F19.19, F19.20, F19.21, F19.220, F19.221, F19.222, F19.229, F19.230, F19.231, F19.232, F19.239, F19.24, F19.250, F19.251, F19.259, F19.26, F19.27, F19.280, F19.281, F19.282, F19.288, F19.29, O99.320, O99.321, O99.322, O99.323, O99.324, O99.325
Psychotic Disorders	F06.0, F06.1, F06.2, F06.30, F06.33, F11.150, F11.151, F11.159, F11.250, F11.251, F11.259, F11.950, F11.951, F11.959, F12.150, F12.151, F12.159, F12.250, F12.251, F12.259, F12.950, F12.951, F12.959, F13.150, F13.151, F13.159, F13.250, F13.251, F13.259, F13.950, F13.951, F13.959, F14.150, F14.151, F14.159, F14.250, F14.251, F14.259, F14.950, F14.951, F14.959, F15.150, F15.151, F15.159, F15.250, F15.251, F15.259, F15.950, F15.951, F15.959, F16.150, F16.151, F16.159, F16.250, F16.251, F16.259, F16.950, F16.951, F16.959, F18.150, F18.151, F18.159, F18.250, F18.251, F18.259, F18.950, F18.951, F18.959, F19.150, F19.151, F19.159, F19.250, F19.251, F19.259, F19.950, F19.951, F19.959, F20.0, F20.1, F20.2, F20.3, F20.5, F20.81, F20.89, F20.9, F21, F22, F23, F24, F25.0, F25.1, F25.8, F25.9, F28, F29, F30.10, F30.11, F30.12, F30.13, F30.2, F30.3, F30.4, F30.8, F30.9, F31.0, F31.10, F31.11, F31.12, F31.13, F31.2, F31.30, F31.31, F31.32, F31.4, F31.5, F31.60, F31.61, F31.62, F31.63, F31.64, F31.70, F31.71, F31.72, F31.73, F31.74, F31.75, F31.76, F31.77, F31.78, F31.81, F31.89, F31.9, F32.4, F32.5, F33.40, F33.41, F33.42, F34.0, F34.8, F34.81, F34.89, F34.9, F39, F44.89, F84.3
Obstructive Sleep Apnea	G47.33
Peptic Ulcer Disease, with and without bleeding	K25.0, K25.1, K25.2, K25.3, K25.4, K25.5, K25.6, K25.7, K25.9, K26.0, K26.1, K26.2, K26.3, K26.4, K26.5, K26.6, K26.7, K26.9, K27.0, K27.1, K27.3, K27.4, K27.5, K27.6, K27.7, K27.9, K28.0, K28.1, K28.2, K28.3, K28.4, K28.5, K28.6, K28.7, K28.9
Valvular Disease	A18.84, A32.82, A39.51, A52.03, B33.21, B37.6, I01.1, I01.8, I01.9, I02.0, I05.0, I05.1, I05.2, I05.8, I05.9, I06.0, I06.1, I06.2, I06.8, I06.9, I07.0, I07.1, I07.2, I07.8, I07.9, I08.0, I08.1, I08.2, I08.3, I08.8, I08.9, I09.1, I09.89, I33.0, I33.9, I34.0, I34.1, I34.2, I34.8, I34.9, I35.0, I35.1, I35.2, I35.8, I35.9, I36.0, I36.1, I36.2, I36.8, I36.9, I37.0, I37.1, I37.2, I37.8, I37.9, I38, I39, M32.11, Q22.0, Q22.1, Q22.2, Q22.3, Q22.4, Q22.5, Q22.6, Q22.8, Q22.9, Q23.0, Q23.1, Q23.2, Q23.3, Q23.4, Q23.8, Q23.9, T82.01XA, T82.01XD, T82.01XS, T82.02XA, T82.02XD, T82.02XS, T82.03XA, T82.03XD, T82.03XS, T82.09XA, T82.09XD, T82.09XS, T82.221A, T82.221D, T82.221S, T82.222A, T82.222D, T82.222S, T82.223A, T82.223D, T82.223S, T82.228A, T82.228D, T82.228S, T82.6XXA, T82.6XXD, T82.6XXS, Z95.2, Z95.3, Z95.4
Atrial Fibrillation and Flutter	I480, I481, I482, I483, I484, I485, I486, I487, I488, I489
Prior Permanent Pacemaker	Z95.0
Prior Implantable Cardioverter-Defibrillator	Z95.810
Previous Cardiac Surgery	Z95.2, Z98.890
Conduction Diseases	I44.0, I44.1, I44.2, I44.3, I44.4, I44.5, I44.6, I44.7, I44.8, I44.9, I45.0, I45.1, I45.2, I45.3, I45.4, I45.5, I45.6, I45.7, I45.8, I45.9
Acid-base and Electrolyte Disturbances	E8350, E8351, E8352, E8359, E8340, E8341, E8342, E8349, E8339, E8339, E87
Gestational Diabetes	O24.4
Pre-Eclampsia	O14.0, O14.1, O14.9, O14.90, O14.91, O14.92, O14.93, O14.94, O14.95, O11, O11.1, O11.2, O11.3, O11.4, O11.5, O11.9, O14.00, O14.02, O14.03, O14.04, O14.05, O14.10, O14.12, O14.13, O14.14, O14.15
HELLP Syndrome	O14.2, O13.20, O14.22, O14.23, O14.24, O14.25
Eclampsia	O15, O15.0, O15.00, O15.02, O15.03, O15.1, O15.2, O15.9
Cesarean Delivery	10D00Z0, 10D00Z1, 10D00Z2
Antepartum or Postpartum Hemorrhage	O72, O72.1, O72.2
Acute Heart Failure	I50.21, I50.31, I50.41, I50.23, I50.33, I50.43
Acute Kidney Injury	N17.0, N17.1, N17.2, N17.8, N17.9, N19
Cardiogenic Shock	R57.0
Ventricular Arrhythmia	I47.2, I49.01, I49.02
Cardioversion	5A2204Z
Sudden Cardiac Arrest	I46.2, I46.8, I46.9
Intra-cardiac thrombus	I51.3, I23.6
Embolic Stroke	I63.0, I63.1, I63.3, I63.4
Thromboembolic Events	I63.0, I63.1, I63.3, I63.4, I63.6, I74.0, I74.1, I74.2, I74.3, I74.4, I74.5, I74.6, I74.7, I74.8, I74.9, O88.21, O88.212, O88.213, O88.219, I82.A1, I82.A2, I82.B1, I82.B2, I82.C1, I82.C2, I23.6, I24.0, I51.3, T82.867, T82.868
CRTP ^f^	0JH607Z, 0JH637Z, 0JH807Z, 02HL4JZ, 02HL3JZ, 02HL0JZ, 02H44JZ, 02H43JZ, 02H40JZ
CRTD ^g^	0JH609Z, 0JH639Z, 0JH839Z, 02HL4KZ, 02HL3KZ, 02HL0KZ, 02H43KZ, 02H40KZ
Implantable Cardioverter Defibrillator	0JH608Z, 0JH638Z, 0JH808Z, 0JH838Z, O2H43JZ, O2H43JZ, 02H43JZ, 02H43MZ, 02H63JZ, 02HJ3KZ, 02HK3KZ, 02HN0KZ, 02HN4KZ, 02H60KZ, 02H64KZ
Permanent Pacemaker	OJH604Z, 0JH634Z, 0JH804Z, 0JH834Z, 0JH605Z, 0JH6357, 0JH805Z, 0JH835Z, 0JH606Z, 0JH636Z, 0JH806Z, 0JH836Z, 02HK4JZ, 02HK0JZ
Mechanical Circulatory Support	5A0221D, 02HA3RJ, 02HA3RZ, 02HA0RZ, 02PA3RZ, 5A02210, 5A1522F, 5A15A2F, 5A1522G, 5A02116, 5A02216, 02HA0QZ, 02HA0RZ, 02HA3QZ, 02HA3RZ, 02HA4QZ, 02RL0JZ, 02RL4JZ, 02UA0JZ, 02UA3JZ
